# Blood Lead Mediates the Relationship between Biological Aging and Hypertension: Based on the NHANES Database

**DOI:** 10.3390/nu16132144

**Published:** 2024-07-05

**Authors:** Cuixiao Wang, Jinming Su, Jinmiao Li, Wenfei Wei, Zongxiang Yuan, Rongfeng Chen, Wudi Wei, Youjin Huang, Li Ye, Hao Liang, Junjun Jiang

**Affiliations:** 1 Guangxi Crucial Laboratory of AIDS Prevention and Treatment, School of Public Health, Guangxi Medical University, Nanning 530021, Chinayeli@gxmu.edu.cn (L.Y.); lianghao@gxmu.edu.cn (H.L.); 2 Collaborative Innovation Centre of Regenerative Medicine and Medical BioResource Development and Application Co-Constructed by the Province and Ministry, Life Science Institute, Guangxi Medical University, Nanning 530021, China

**Keywords:** hypertension, biological aging, blood lead, mediation analysis

## Abstract

Hypertension remains a major global public health crisis due to various contributing factors, such as age and environmental exposures. This study delves into exploring the intricate association between biological aging, blood lead levels, and hypertension, along with examining the mediating role of blood lead levels in the relationship between biological aging and hypertension. We analyzed data from two cycles of the NHANES, encompassing 4473 individuals aged 18 years and older. Our findings indicate that biological aging potentially escalates the risk of hypertension and the incidences of systolic blood pressure (SBP) and diastolic blood pressure (DBP) abnormalities. Utilizing weighted quantile sum (WQS) and quantile g-computation (QGC) model analyses, we observed that exposure to heavy metal mixtures, particularly lead, may elevate the likelihood of hypertension, SBP, and DBP abnormalities. Further mediation analysis revealed that lead significantly mediated the relationship between biological aging and hypertension and between biological aging and SBP abnormalities, accounting for 64% (95% CI, 49% to 89%) and 64% (95% CI, 44% to 88%) of the effects, respectively. These outcomes emphasize the criticality of implementing environmental health measures.

## 1. Introduction

Hypertension stands as a significant global health concern. According to the 2023 “Global Report on Hypertension”, the number of people with hypertension globally increased from 650 million in 1990 to 1.3 billion in 2019. It is estimated that hypertension causes 7.7 to 10.4 million deaths annually [1]. Hypertension not only leads to symptoms such as headaches and dizziness but, more importantly, it can cause serious conditions like myocardial infarction, stroke, heart failure, and premature death. The high prevalence and mortality rates of hypertension result in significant physical harm to patients and impose a heavy burden on society and families [2,3]. Various factors, including lifestyle [2], age [2,4], genetics [2], physical inactivity [5], diet [2,6], and environmental exposures [7], influence the occurrence of hypertension. Evidence indicates that pollutants, such as PM2.5, lead, and cadmium, also increase the prevalence of hypertension [7,8]. Despite controlling for many risk factors, hypertension remains highly prevalent, and its exact causes are still not fully understood.

Aging is characterized by physiological slowing, a decreased regenerative capacity, and an inability to maintain cellular homeostasis. It significantly increases the likelihood of developing a variety of chronic illnesses, including diabetes mellitus, cardiovascular disease (CVD), and hypertension [9]. According to a survey, over 70% of individuals aged 65 years and older in the United States have hypertension, with its prevalence increasing with age [10]. Older adults are also more sensitive to the adverse effects of environmental exposures due to age-related declines in organ functions, including kidney and liver functions [11]. This decline affects the body’s ability to absorb and metabolize various toxic substances, including heavy metals like lead and mercury. Additionally, even individuals of the same chronological age can have varying susceptibilities to age-related diseases, indicating that chronological age does not always accurately reflect biological aging [12].

Lead (Pb), cadmium (Cd), mercury (Hg), manganese (Mn), and selenium (Se) are widespread environmental heavy metal contaminants [13]. Organisms are exposed to these metals through various pathways, including air pollution, household wastewater, cosmetics, and food consumption [14]. There is increasing evidence that the accumulation of heavy metals in mammals is linked to a variety of health problems, such as cardiovascular disease, metabolic syndrome, and cancer [15]. Lead is a ubiquitous environmental toxicant, with primary sources being dust, soil, paint, and water [13]. Several animal studies have demonstrated the induction of hereditary hypertension by chronic exposure to low levels of lead [16]. Cross-sectional studies have indicated a significant correlation between lead and the prevalence of hypertension in the United States [17], Brazil [18], South Korea [19], and China [20]. However, in urban Haiti, although higher lead levels were associated with higher SBP and DBP, there was no statistically significant association found between lead and hypertension [21]. Yang’s study did not find any association between ambulatory blood pressure and blood lead [22]. In previous studies based on the databases of NHANES III and NHANES 2003–2010, blood lead and hypertension were also not found to be related [23,24]. Therefore, the relationship between blood lead and hypertension is uncertain.

Following exposure to lead, the kidneys are vulnerable to lead poisoning and serve as the main organ for its excretion. Aging often accompanies a decline in kidney function, which reduces the efficiency of lead excretion [11], making lead more likely to accumulate in the body. In the present study, we hypothesized that lead, as a common environmental pollutant, may mediate the effects of aging on blood pressure. Additionally, exposure to multiple heavy metals in the natural environment usually co-occurs, and their effects can be superimposed, synergistic, or antagonistic. Therefore, considering the context of heavy metal mixture exposure, we explored potential associations between heavy metal mixtures and blood pressure using a weighted quantile sum (WQS) and the quantile g-computation (QGC) model. We identified lead as a significant positive exposure. Furthermore, logistic regression was employed to explore the effects of aging and blood lead on blood pressure. Finally, mediation analysis was employed to investigate the role of blood lead in mediating the impact of aging on blood pressure.

## 2. Methods

### 2.1. Study Population

**Figure 1:**
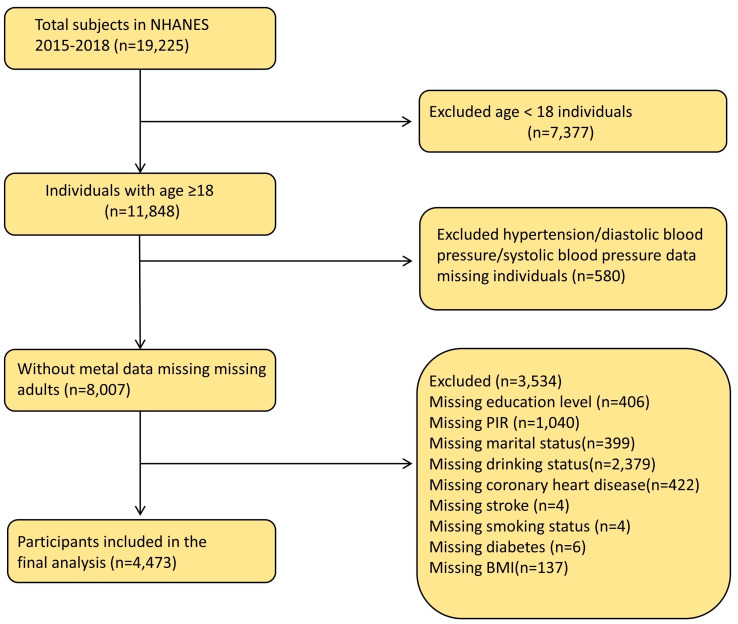
Flow chart of the population included in this study.

The NHANES data collection involved interviews, home screenings, and physical examinations. All data can be obtained from the NHANES website. Since these data already received approval from the NHANES Medical Ethics Committee, no further ethical clearance was necessary. This study selected individuals from two cycles (2015–2018) of the NHANES database. We chose participants aged 18 years or older who underwent blood heavy metal testing, excluding those younger than 18 years (n = 7377) or those who lacked data concerning heavy metals (n = 10,818), hypertension, DBP and SBP (n = 580), or covariate data (n = 3534). We included 4473 US adults in our study. Figure 1 shows the flow chart for excluding the study population.

### 2.2. PhenoAge and PhenoAgeAccel Assessments

PhenoAge, developed by Levine et al., is a metric used to estimate biological age. It incorporates both chronological age and nine biomarkers, including albumin and C-reactive protein, mean cell volume, white blood cell count, and alkaline phosphatase, among others. PhenoAgeAccel is calculated as the difference between the PhenoAge and the chronological age. A higher PhenoAgeAccel indicates greater biological aging [25]. The specific calculation for the formula of PhenoAge and PhenoAgeAccel is shown in Supplementary Figure S1.

### 2.3. Blood Pressure Measurement and Definitions

Trained personnel and/or physicians conducted the blood pressure measurements at the mobile testing center. After resting quietly in a seated position for 5 min, three consecutive blood pressure readings were recorded. Participants were considered to have high blood pressure if they gave an affirmative answer to the following questions: “Have you ever been told by a doctor or other health professional that you have hypertension, also called high blood pressure?”; “Are you now taking prescribed medicine for high blood pressure?”; or if they had a high biological measurement value (systolic blood pressure ≥ 140 mm Hg and/or diastolic blood pressure ≥ 90 mm Hg) [26]. According to the American Heart Association, we used 120 and 80 mm Hg as the cutoff values for abnormal systolic and diastolic blood pressures, respectively [27]. 

### 2.4. Heavy Metals Assessments

Whole blood samples were stored at −30 °C and subsequently transported for analysis. Heavy metal concentrations in the blood were determined using inductively coupled plasma mass spectrometry as part of NHANES. The results were reported in μg/dL. To ensure the reliability of the findings, any measurements below the limit of detection were excluded [26].

### 2.5. Study Covariates

In this study, we incorporated several covariates, including sex (male and female), age (18–39, 40–59, and ≥60 years), race (White, Black, and others), income status based on the poverty impact ratio (PIR) (<2 and ≥2), smoking status (smoking at least 100 cigarettes in a lifetime or not), drinking status (defined as an individual who consumed a minimum of 12 alcoholic drinks per year (NHANES 2003–2016) or consumed alcohol at least once a month (NHANES 2017–2018)), education level (below high school, high school, and above high school), marital status (single/divorced/widowed/separated or married/cohabited), body mass index (BMI) (<25, 25–30, and ≥30), and history of diseases (including diabetes and coronary heart disease (CHD)). Data about all these covariates were obtained via standardized questionnaires or instrumental measurement. Cardiovascular diseases and diabetes were defined as a positive answer to the question, “Have you ever been told you had coronary heart disease/stroke/diabetes”?

### 2.6. Statistical Analyses

Continuous variables are presented as the mean with standard deviation, and categorical variables are presented as frequencies and percentages. Chi-squared tests were used for categorical variables. Blood heavy metal concentrations were logarithmically transformed and categorized into quartiles. Kendall’s tau correlation was used to assess the relationships among the concentrations of the five heavy metals. First, we employed both unadjusted and adjusted logistic regression models to examine the relationships between PhenoAgeAccel and hypertension, SBP, and DBP. The crude model was unadjusted, while the adjusted model was controlled for age, sex, race, BMI, PIR, education level, smoking, drinking status, marital status, diabetes, coronary heart disease (CHD), stroke, and blood metal. In this study, hypertension was used as the main dependent variable analyzed. Additionally, in the analysis of PhenoAgeAccel and hypertension, we performed subgroup analyses by stratifying the population by age, sex, race, BMI, PIR, et al., to assess the sensitivity and interactions within different subgroups.

Second, to evaluate the aggregate and individual effects of heavy metal mixtures on hypertension, DBP, and SBP and to identify the main blood heavy metal variables contributing to the mode, we employed bootstrapping with 10,000 iterations to construct WQS indexes in a positive direction [28]. When the WQS index showed significance, we analyzed the corresponding weights to determine the relative contribution of each heavy metal to the prevalence of hypertension, DBP, and SBP. In the QGC analysis, the weights assigned to heavy metals indicated their relative positive or negative contributions to the overall exposure mixture, with each exposure range set between 0 and 1 [28]. Additionally, after identifying blood lead as the variable with the largest positive contribution to both models, we performed an RCS analysis using InPb with hypertension, DBP, and SBP. We also analyzed the relationships between blood lead and hypertension, DBP, and SBP using logistic regression with both crude and adjusted models, where the crude model was unadjusted for covariates, and the adjusted model was adjusted for all covariates, as described previously.

Third, to explore the mediating role of blood lead between PhenoAgeAccel and hypertension, DBP, and SBP, we conducted a mediation analysis using nonparametric bootstrapping (n = 1000) to investigate both direct and indirect relationships, as well as to assess the magnitude of the mediating effects [29]. The crude model was unadjusted for covariates, and the adjusted model was adjusted for all covariates and other heavy metals to explore stability. The statistical analyses were performed using R version 4.3.0. A two-sided *p*-value of <0.05 was considered statistically significant in all analyses.

## 3. Result

### 3.1. Baseline Characteristic

The characteristics of the participants are shown in Table 1. A total of 4473 participants, aged 20–80 years, had a mean age of 49.50 ± 17.21 years. Among them, 1633 (36.51%) suffered from hypertension, 1044 (23.34%) had DBP abnormality, 2475 (55.33%) had SBP abnormality, and 1812 (40.50%) exhibited PhenoAgeAccel. Generally, participants with hypertension, abnormal SBP, and DBP were more likely to be older, have comorbid CHD, have higher BMI, and have higher blood lead concentrations. 

**Figure 2:**
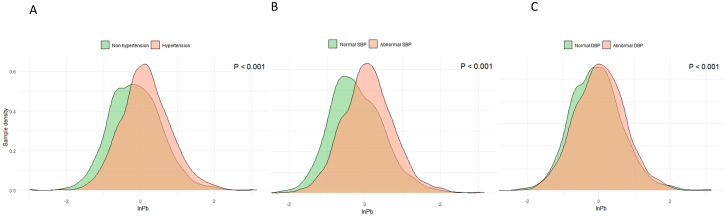
Sample density curves of lead concentrations among participants. (**A**) nonhypertension and hypertension; (**B**) normal SBP and abnormal SBP; (**C**) normal DBP and abnormal DBP.

Correlation analyses revealed significant correlations between InPb and InHg, InPb and InCd, and InHg and InSe (Supplementary Figure S2). Sample density curves showed that patients with high blood lead tended to have hypertension, SBP, and DBP abnormalities, compared to the general population (Figure 2).

### 3.2. Association between PhenoAgeAccel and Hypertension, SBP, and DBP

**Figure 3:**
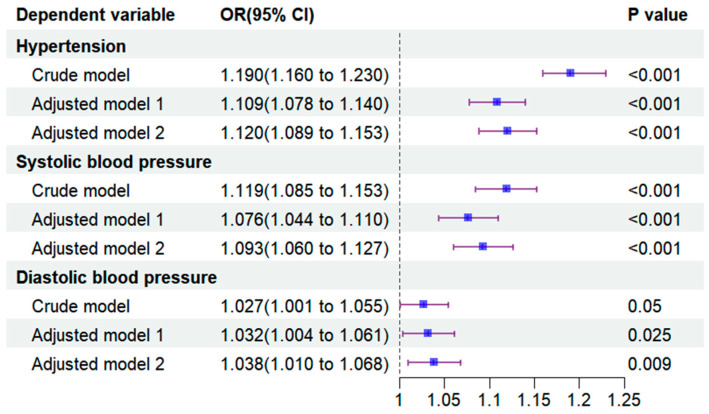
Association between PhenoAgeAccel and hypertension, SBP, and DBP.

As shown in Figure 3, PhenoAgeAccel was significantly correlated with hypertension, SBP, and DBP. The crude model did not adjust for variables, while adjusted model 1 accounted for all covariates, and model 2 additionally adjusted for blood metal levels. In adjusted model 2, PhenoAgeAccel was associated with significant increases in hypertension risk (OR 1.12, 95% CI 1.089 to 1.153), SBP abnormalities (OR 1.093, 95% CI 1.06 to 1.127), and DBP abnormalities (OR 1.038, 95% CI 1.01 to 1.068).

Subgroup analyses were conducted based on age, sex, BMI, PIR, education level, smoking status, drinking status, CHD, stroke, and diabetes. The results consistently showed that PhenoAgeAccel was associated with hypertension across all subgroups. Notably, sex had an interaction with PhenoAgeAccel in relation to hypertension, whereas there was no significant interaction between PhenoAgeAccel and other factors (Supplementary Figure S3). 

### 3.3. Association between Co-Exposure of Blood Metals and Hypertension, SBP, and DBP

**Figure 4:**
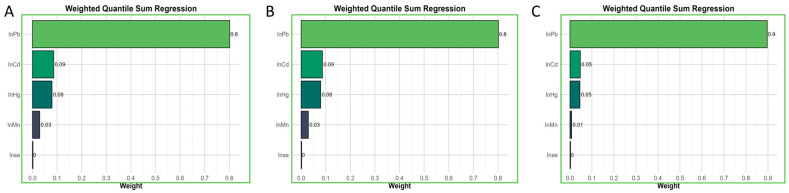
The weights of each blood heavy metal in positive WQS model regression. (**A**) Hypertension; (**B**) SBP; (**C**) DBP.

The WQS and QGC models were used to investigate the potential effects of co-exposure to blood metals and the determination of major metal exposure. Our findings indicated that co-exposure to blood metals had a significant impact on hypertension, SBP, and DBP. In the adjusted model, which adjusted for all covariates, the WQS index of mixed metals was positively associated with hypertension (OR 1.35, 95% CI 1.24 to 1.48), SBP (OR 1.53, 95% CI 1.39 to 1.68), and DBP (OR 1.28, 95% CI 1.13 to 1.46) (Table 2). Additionally, in the analysis of hypertension, SBP, and DBP, the highest weighted metal in the WQS models was lead, at 80%, 80%, and 90%, respectively (Figure 4). Similarly, the QGC model also showed a significant and positive association of mixed metals with hypertension, SBP, and DBP (Table 2, Supplementary Figure S4).

### 3.4. Blood Lead and Hypertension, SBP, and DBP in the Logistic Regression Model

**Figure 5:**
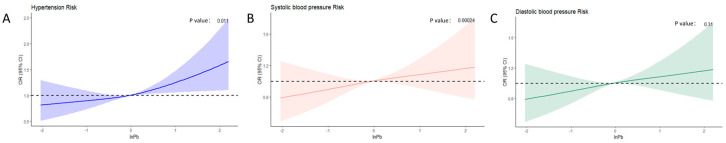
RCS model of blood lead levels and risks of hypertension, SBP, and DBP. (**A**) Hypertension; (**B**) SBP; (**C**) DBP.

To evaluate the potential link between the quartiles of log-transformed lead metal concentrations and hypertension, SBP, and DBP abnormalities, we employed both crude and adjusted models. The crude model did not adjust for variables, while the adjusted model adjusted for all covariates and other blood metals. The Supplementary Table S1 shows that compared with baseline PhenoAgeAccel quartile 1, lead was significantly correlated with an elevated prevalence of hypertension and SBP abnormalities in Q2–Q4 in both the crude model and the adjusted model. Additionally, we observed an increased risk of DBP abnormalities for lead in Q3–Q4 in both the crude and adjusted models. Figure 5 shows that the RCS model indicated a significant nonlinear association between blood lead levels and the risks of hypertension and SBP.

### 3.5. The Mediation Effects of Blood Lead in the Relationships of PhenoAgeAccel with Hypertension, SBP, and DBP

**Figure 6:**
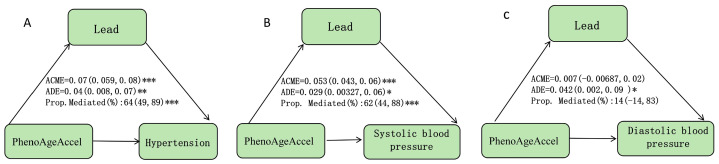
Mediation effects of estimated blood lead on the associations of PhenoAgeAccel with hypertension, SBP, and DBP. Notes: ACME, average causal mediation effects (indirect effect); ADE, average direct effects. * *p* < 0.05, ** *p* < 0.01, and *** *p* < 0.001. (**A**) Hypertension; (**B**) SBP; (**C**) DBP.

Significant mediation effects of lead were noted in the associations between PhenoAgeAccel and hypertension and SBP. In the adjusted model, which accounted for all covariates and other blood metals, the mediated proportions (%) of lead in the relationships of PhenoAgeAccel with hypertension and SBP were 64% (95% CI: 49%, 89%) and 64% (95% CI: 44%, 88%), respectively (Figure 6).

## 4. Discussion

Aging has become a significant global concern, characterized by a continuous decline in physiological functions and loss of capabilities, leading to systemic dysfunctions, such as telomere attrition, epigenetic alterations, protein homeostasis disruption, mitochondrial dysfunction, and cellular senescence [10]. These dysfunctions can result in diseases like hypertension, diabetes, and neurodegenerative conditions. Consequently, aging is a major risk factor for hypertension. In the medical field, individuals of the same age show considerable variability in physiological markers and disease susceptibility [30]. Among various biological aging algorithms, PhenoAge has demonstrated the highest sensitivity to aging [31]. We calculated PhenoAge and incorporated PhenoAgeAccel, a composite index characterizing the rate of aging. Our research found that aging increases the risk of hypertension and abnormalities in diastolic and systolic blood pressures. Aging results in several structural and functional changes in the arterial vasculature. It may be related to the specific underlying mechanisms, including mechanical hemodynamic changes, arterial stiffness, neurohormonal and autonomic dysregulation, and the aging kidney [32]. The aging changes in the kidney are associated with increased salt sensitivity, which prompts vasoconstriction and vascular resistance [4]. This chronic cellular damage and dysfunction are thought to at least partially contribute to physiologic dysfunction and the development of hypertension.

Our study also found that blood lead concentrations showed a slow increase with age and were associated with age and biological aging (Supplementary Figures S5 and S6). In addition, we found that lead mediates the relationship between aging and hypertension. Over recent decades, the harmful effects of low-level lead exposure have gained increasing attention. Lead enters the body mainly through inhalation or ingestion; it readily passes the air–blood barrier and is distributed systemically via the bloodstream. Lead is a cumulative toxicant, primarily stored in bones and red blood cells [33]. In adults, lead concentrations in bones increase with age, sometimes by up to ten times [34]. The respiratory tract is the body’s first line of defense against respiratory pollutants, enabling an appropriate regulatory response to persistent environmental exposures, and pulmonary immune effector cells ensure a robust response to foreign harmful substances [35]. The intestine, as a dynamic barrier to external exposures, is involved in many physiological processes, including immunomodulation. Aging affects the homeostasis of intestinal epithelial cells and the development of other cells, weakening the barrier function of the intestine [36]. This disequilibrium extends to the lung microenvironments and the respiratory immune interface, leading to the increased absorption of harmful substances [35,36], such as lead, as observed in our study. The kidney is the organ where lead is most widely distributed in the human body, followed by the liver [37]. Aging impairs renal functions, including decreased glomerular filtration rate and glomerulosclerosis, resulting in the reduced metabolism and excretion of lead [38]. These factors may contribute to the increased accumulation of lead in the aging population.

Heavy metals such as lead, cadmium, mercury, manganese, and others are prevalent in both residential and occupational settings, leading to regular exposure in daily life [39]. The impact of heavy metals on blood pressure may be associated with impaired kidney function, inflammation, and oxidative stress [40,41]. Our study aligns with findings from Qu et al., indicating a positive association between blood levels of heavy metals and hypertension. Specifically, lead emerged as the predominant contributor, compared to other metals [42]. Our study found that blood lead may increase the risk of developing hypertension and SBP and DBP abnormalities, supporting the idea that lead may increase the risk of developing hypertension. Chronic lead exposure has been linked to deleterious effects on various organs, contributing to damage through multiple pathways. A previous study found that teenagers and elderly individuals living in high lead exposure areas had significantly higher levels of urinary oxidative stress markers [43]. Oxidative stress has been identified as a potential contributor to vascular dysfunction and target organ damage. Lead, a non-redox-active metal, is associated with oxidative stress and may cause endothelial dysfunction. Lead reduces NO bioavailability, impairs the antioxidant system, and increases the generation of reactive oxygen species (ROS). Lead also depletes cellular antioxidant reserves by selectively binding to sulfur-containing antioxidants and enzymes, thereby generating ROS [44]. Cumulative evidence from human and animal studies suggests that ROS plays a crucial role in regulating endothelial cell function and vascular remodeling [33]. Furthermore, lead exposure has been observed to cause telomere shortening and lipid disturbances, which may play important roles in the pathophysiological processes involved in the development of hypertension [45]. As a divalent cation, lead also affects various signaling pathways, impacting vascular resistance and increasing blood pressure [33]. Lead also reduces (NO) and guanylate cyclase production in blood vessels, leading to vascular remodeling and inhibiting vascular relaxation [46]. Additionally, complications of hypertension include kidney damage [6], which may impair the kidneys’ ability to excrete heavy metals, potentially leading to the increased accumulation of lead and an elevated risk of hypertension. More studies are needed to explore these mechanisms to identify new targets for understanding the molecular mechanisms of lead toxicity. 

Our study found that both aging and lead exposure increases the risk of hypertension, with lead acting as a mediator in this relationship. To reduce hypertension incidence, lifestyle interventions, such as increased exercise, reduced food intake, and obesity, can mitigate phenotypic aging and maintain a healthy lifespan [47]. Measures taken by government departments to control lead emissions, recycle lead-acid batteries, conduct environmental lead testing, ensure safe drinking water, and provide good occupational protection are crucial [48].

This study has several strengths: First, the data were obtained from a large, representative population sample. Second, it is the first study to assess the mediating effect of blood lead in the relationship between biological aging and hypertension. Third, we employed multiple statistical methods and adjusted for potential confounders to enhance the robustness and reliability of our findings. However, this study has some limitations. First, due to its cross-sectional design, we could not establish causality between exposure and outcome. Second, we used blood lead levels as the study variable and did not compare them with lead levels in bones, which may have affected the precision of our results.

## 5. Conclusions

Our study found that biological aging and blood-mixed heavy metals are associated with the risk of developing hypertension, with lead contributing the most to the positive effect of blood-mixed heavy metals on hypertension. Lead also plays a mediating role in the relationship between biological aging and hypertension. Therefore, hypertension can be reduced by addressing these two perspectives.

## Figures and Tables

**Table 1:** Baseline characteristics of participants (n = 4473).

Characteristics	n (%)	*p* Value		
		Hypertension	DBP	SBP
Sex				
male	2247 (50.2)	0.104	<0.001	<0.001
female	2226 (49.8)			
Diabetes				
No	3705 (82.8)	<0.001	0.093	<0.001
Yes	768 (17.2)			
Drinking status				
No	806 (18.0)	0.824	<0.001	0.388
Yes	3667 (82.0)			
Age				
18–39	1490 (33.3)	<0.001	<0.001	<0.001
40–59	1492 (33.4)			
≥60	1491 (33.3)			
Race				
White	1669 (37.3)	0.27	<0.001	0.454
Black and others	2804 (62.7)			
Education level				
Below high school	809 (18.1)	0.006	0.162	<0.001
High school	1033 (23.1)			
Above high school	2631 (58.8)			
Marital status				
Single/divorced/widowed/separated	1762 (39.4)	0.158	0.263	0.233
Married/cohabited	2711 (60.6)			
PIR				
<2	2047 (45.8)	0.313	0.616	0.907
≥2	2426 (54.2)			
CHD				
No	4296 (96.0)	<0.001	0.015	<0.001
Yes	177 (4.0)			
Stroke				
No	4296 (96.0)	<0.001	0.218	<0.001
Yes	177 (4.0)			
Smoking status				
No	2492 (55.7)	<0.001	0.897	<0.001
Yes	1981 (44.3)			
BMI (kg/m^2^)				
<25	1158 (25.9)	<0.001	0.001	<0.001
25–30	1410 (31.5)			
≥30	1905 (42.6)			
InPb				
Q1	1141 (25.5)	<0.001	<0.001	<0.001
Q2	1101 (24.6)			
Q3	1130 (25.3)			
Q4	1101 (24.6)			
InCd				
Q1	1121 (25.1)	<0.001	0.167	0.024
Q2	1121 (25.1)			
Q3	1121 (25.1)			
Q4	1110 (24.8)			
InHg				
Q1	1137 (25.4)	0.204	0.962	0.044
Q2	1112 (24.9)			
Q3	1111 (24.8)			
Q4	1113 (24.9)			
InMn				
Q1	1122 (25.1)	<0.001	0.526	<0.001
Q2	1120 (25.0)			
Q3	1114 (24.9)			
Q4	1117 (25.0)			
InSe				
Q1	1119 (25.0)	0.191	<0.001	0.352
Q2	1118 (25.0)			
Q3	1119 (25.0)			
Q4	1117 (25.0)			
PhenoAgeAccel				
No	2661 (59.5)	<0.001	0.056	<0.001
Yes	1812 (40.5)			

**Table 2:** Association between the mixture exposure of blood heavy metals and hypertension, SBP, and DBP by WQS and QGC analyses.

Method	Hypertension		SBP		DBP	
	OR (95% Cl)	*p*-Value	OR (95% Cl)	*p*-Value	OR (95% Cl)	*p*-Value
WQS						
Crude model	1.37 (1.27, 1.48)	<0.001	1.59 (1.46, 1.73)	<0.001	1.29 (1.15, 1.46)	<0.001
Adjusted model	1.35 (1.24, 1.48)	<0.001	1.53 (1.39, 1.68)	<0.001	1.28 (1.13, 1.46)	<0.001
QGC						
Crude model	1.317 (1.178, 1.474)	<0.001	1.323 (1.18, 1.483)	<0.001	1.255 (1.105, 1.425)	<0.001
Adjusted model	1.385 (1.226, 1.565)	<0.001	1.352 (1.2, 1.524)	<0.001	1.288 (1.128, 1.471)	<0.001

## Data Availability

All data used in this study are available on the NHANES website (https://wwwn.cdc.gov/nchs/nhanes/Default.aspx, accessed on 11 May 2024).

## Supplementary Materials

The following supporting information can be downloaded at: https://www.mdpi.com/article/10.3390/nu16132144/s1. Supplementary Table S1: Association between blood lead and hypertension, SBP, and DBP. Supplementary Figure S1: The calculation formula of PhenoAge and PhenoAgeAccel. Supplementary Figure S2: Correlation between blood heavy metals. Supplementary Figure S3: Association between PhenoAgeAccel and hypertension, including interactive analysis in subgroups. Supplementary Figure S4: The weights of each blood heavy metal in the positive QGC model regression. Supplementary Figure S5: Correlation between age and blood lead. Supplementary Figure S6: Association between PhenoAgeAccel and blood lead, with the crude model not adjusted for variables, and the adjusted model adjusted for all covariates.
