# Supplementary material for: Blood Lead Mediates the Relationship between Biological Aging and Hypertension: Based on the NHANES Database
Source: Nutrients. 2024 Jul 5;16(13):2144. doi: 10.3390/nu16132144 (PMC11243065; doi:10.3390/nu16132144)
Supplement: Supplementary file 1 [file nutrients-16-02144-s001.zip › Supplementary Table S1.pdf]

Supplementary Table S1:Association between blood lead and hypertension, SBP, and DBP

	Crude Model		Adjusted Model	
	OR (95%Cl)	P value	OR (95%Cl)	P value
DBP				
Q1	Reference		Reference	
Q2	1.02(0.984,1.061)	0.255	1.014(0.976,1.052)	0.4565
Q3	1.051(1.013,1.09)	0.008	1.043(1.004,1.083)	0.03
Q4	1.082(1.042,1.122)	<0.001	1.063(1.022,1.105)	0.002
SBP				
Q1	Reference		Reference	
Q2	1.15(1.104,1.199)	<0.001	1.127(1.082,1.174)	<0.001
Q3	1.289(1.237,1.342)	<0.001	1.244(1.193,1.297)	<0.001
Q4	1.305(1.253,1.360)	<0.001	1.250(1.198,1.305)	<0.001
Hypertension				
Q1	Reference		Reference	
Q2	1.127(1.083,1.172)	<0.001	1.104(1.062,1.146)	<0.001
Q3	1.204(1.158,1.252)	<0.001	1.159(1.115,1.203)	<0.001
Q4	1.249(1.201,1.3)	<0.001	1.224(1.176,1.272)	<0.001
