# Supplementary figures and images for: Blood Lead Mediates the Relationship between Biological Aging and Hypertension: Based on the NHANES Database
Source: Nutrients. 2024 Jul 5;16(13):2144. doi: 10.3390/nu16132144 (PMC11243065; doi:10.3390/nu16132144)

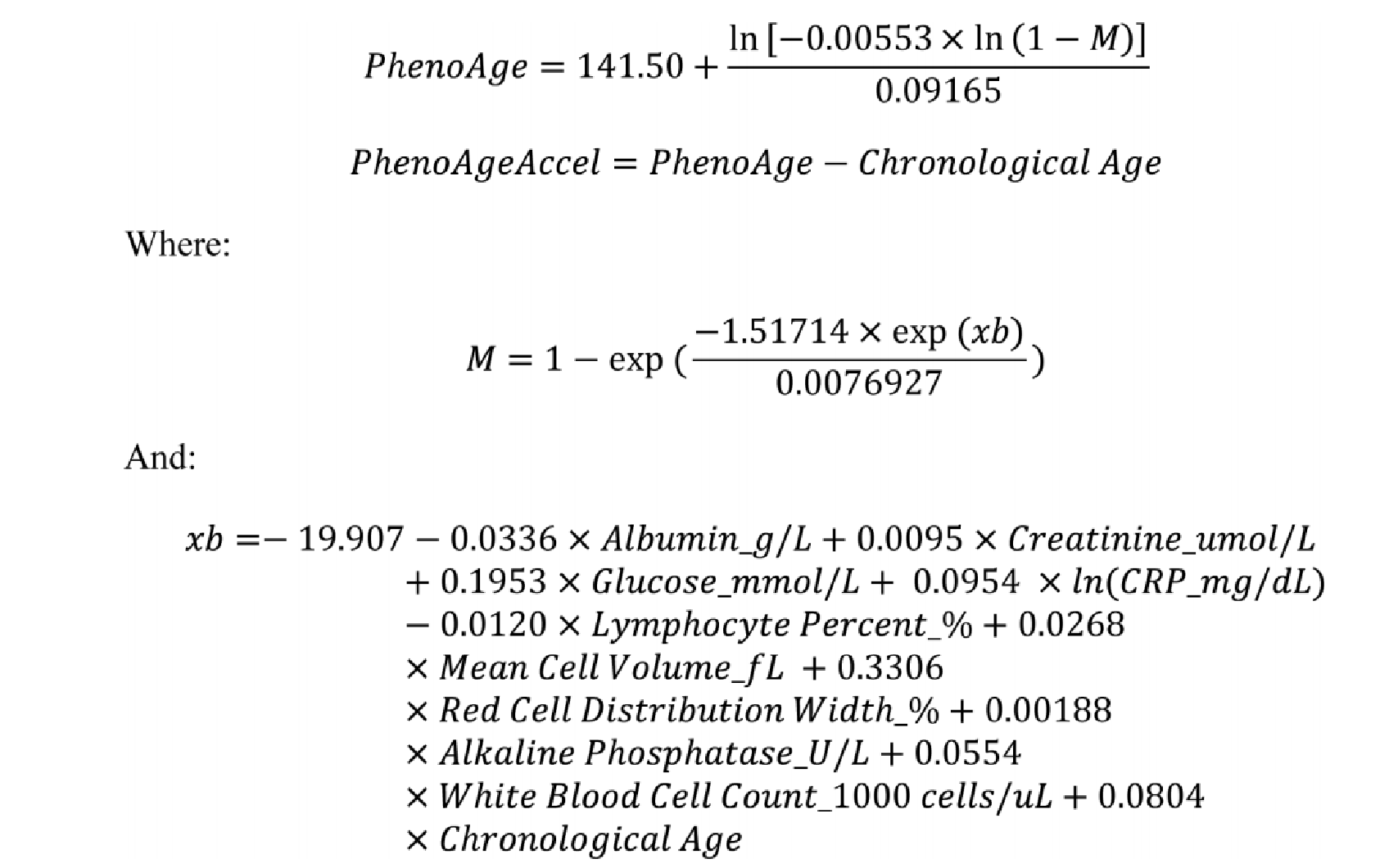

Supplement: Supplementary file 1 [file nutrients-16-02144-s001.zip › Supplementary Fig S1.png]

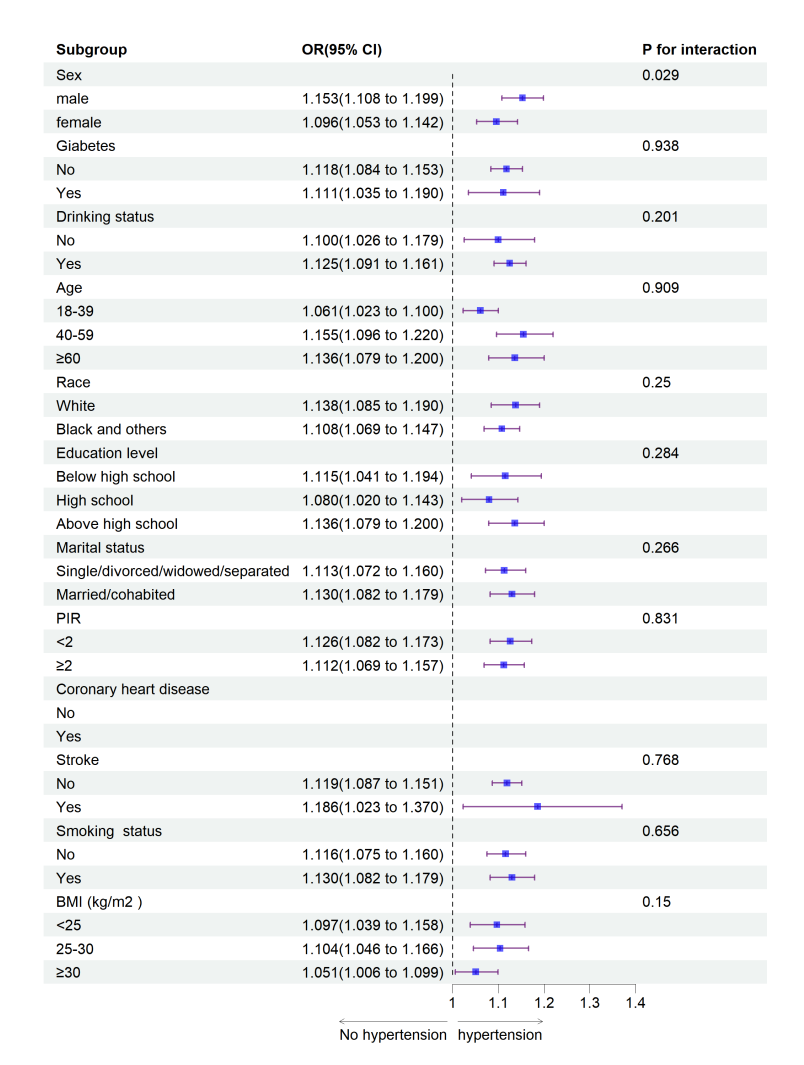

Supplement: Supplementary file 1 [file nutrients-16-02144-s001.zip › Supplementary Fig S3.jpg]

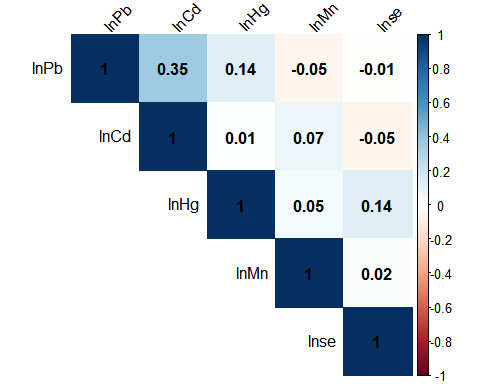

Supplement: Supplementary file 1 [file nutrients-16-02144-s001.zip › Supplementary Fig. S2.png]

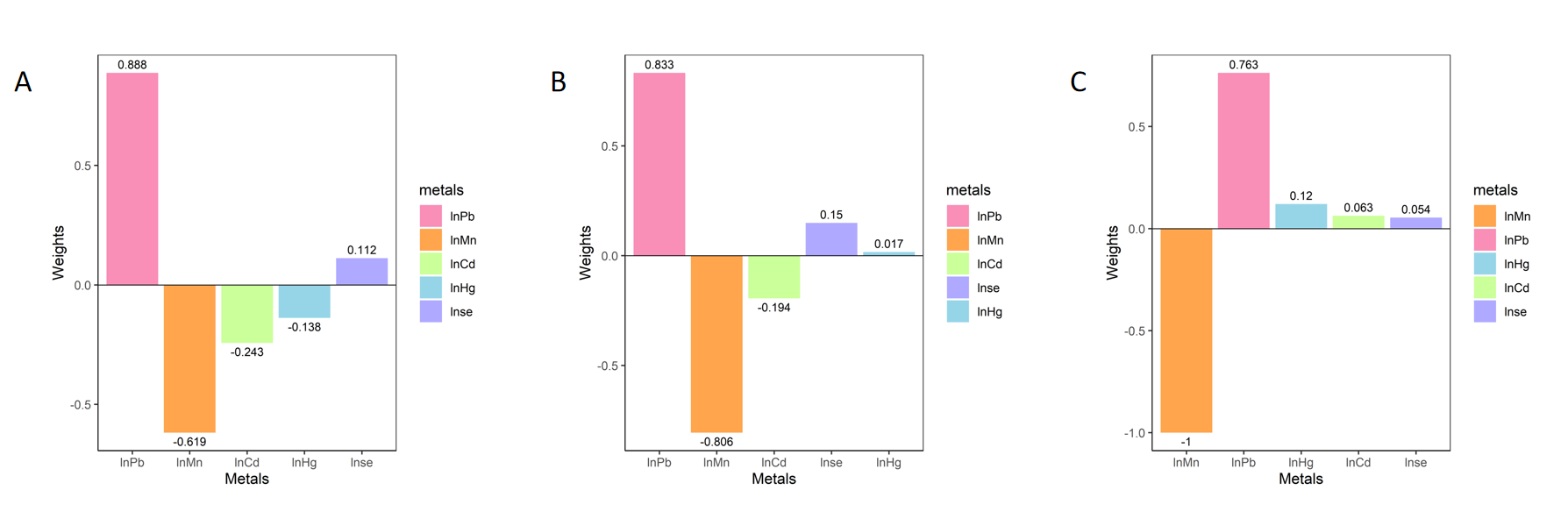

Supplement: Supplementary file 1 [file nutrients-16-02144-s001.zip › Supplementary Fig. S4 .png]

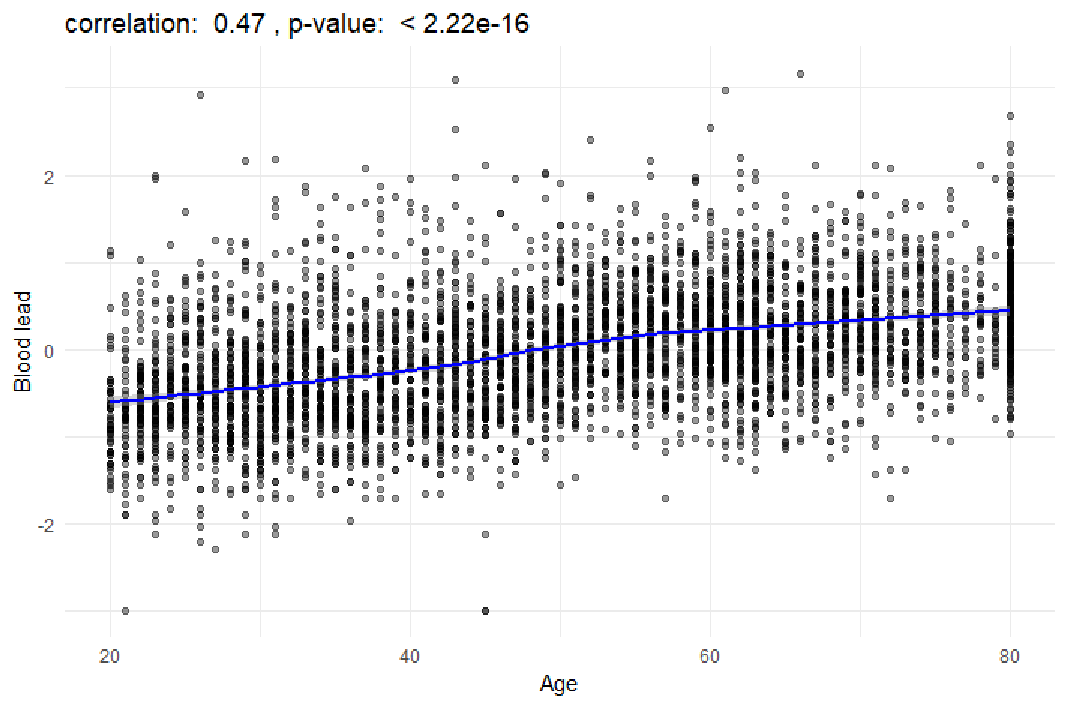

Supplement: Supplementary file 1 [file nutrients-16-02144-s001.zip › Supplementary Fig. S5 .jpg]

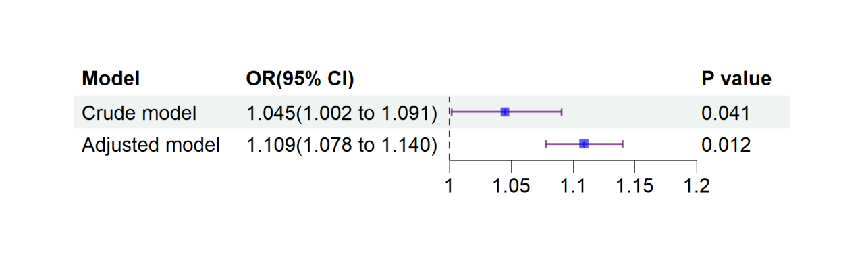

Supplement: Supplementary file 1 [file nutrients-16-02144-s001.zip › Supplementary Fig. S6.jpg]
